# Epidemiological and Genomic Characterization of H5 Subtype Avian Influenza Viruses in Jining City, 2024–2025

**DOI:** 10.3390/pathogens15050521

**Published:** 2026-05-12

**Authors:** Haixia Yang, Yang Zhang, Xiaoyu Wang, Ting Chen, Yongjian Jia, Huixin Dou, Yangbei Jiao, Feifei He, Yajuan Jiang, Boyan Jiao

**Affiliations:** 1 College of Medical Imaging and Laboratory Medicine, Jining Medical University, 133 Hehua Road, Jining 272067, China; haixia_cn@126.com; 2 Department of Laboratory, Yanzhou District Center for Disease Control and Prevention, Jining 272100, China; 15166706416@163.com; 3 Department of Laboratory, Jining Center for Disease Control and Prevention, Jining 272000, China; wangxiaomao5236@163.com (X.W.); yongjianjia4@gmail.com (Y.J.); douhuixin@126.com (H.D.); yangbeijiao@gmail.com (Y.J.); 4 Jining Key Laboratory of Infectious Disease Control and Prevention, Jining 272000, China; 5 Jining Key Laboratory of Precise Identification and Traceability of Emerging and Rare Pathogens, Jining 272000, China; 6 School of Basic Medicine, Jining Medical University, Jining 272067, China; chenting3465@163.com; 7 Computer Information Technology, Northern Arizona University, Flagstaff, AZ 86011, USA; fh335@nau.edu

**Keywords:** H5 subtype avian influenza virus, epidemiological characteristics, whole-genome characterization, clade 2.3.4.4b

## Abstract

**Objective**: The aim of this study was to characterize the epidemiological features and whole-genome characteristics of H5 subtype avian influenza viruses circulating in Jining City during 2024–2025, and to provide scientific evidence for avian influenza prevention and control. **Methods**: A total of 748 poultry-related environmental samples were collected in March, June, September, and December of 2024–2025. Reverse transcription quantitative real-time polymerase chain reaction (RT-qPCR) was used to detect influenza A virus and H5 subtype viral RNA. H5-positive samples were subjected to whole-genome sequencing and analyzed using bioinformatics tools. **Results**: Among the 748 samples, the positivity rate of influenza A virus was 16.04% (120/748), and that of the H5 subtype was 8.16% (61/748). The H5 positivity rate in 2025 (11.88%) was significantly higher than that in 2024 (5.37%). Higher positivity rates were observed in March and December compared to June and September. Twelve complete H5 genomes were obtained, including nine H5N1 and three H5N6 strains. All HA genes belonged to clade 2.3.4.4b. Key mutations related to antigenic drift, replication and adaptation were detected in multiple viral proteins. **Conclusions**: The positivity rate of H5 subtype avian influenza viruses in Jining City showed an increasing trend during 2024–2025, with higher prevalence in winter and spring. The circulating strains predominantly belonged to clade 2.3.4.4b. Antigenic drift-associated mutations in the HA protein were identified in some strains, which may affect vaccine matching. Enhanced surveillance of H5 viruses and regular evaluation of antigenic compatibility between vaccine and circulating strains are recommended to mitigate potential risks posed by viral genetic variation.

## 1. Introduction

Alphainfluenzavirus influenzae, belonging to the Orthomyxoviridae family, represents a major economic threat to the global poultry industry. These enveloped viruses have a segmented RNA genome of negative polarity. In the virion the RNA-dependent RNA polymerase complex is attached to the RNA molecule permitting the RNA translation [1,2]. Certain subtypes of avian influenza viruses can cross species barriers to infect humans and other mammals, posing significant risks to public health [3]. Among them, the H5N1 subtype is one of the most pathogenic avian influenza viruses. It was first identified in poultry in 1996, and the first human infection was reported in Hong Kong, China, in 1997. In 2008, clade 2.3.4.4 emerged and subsequently diversified into eight subclades (2.3.4.4a–2.3.4.4h) [4,5].

H5N1 clade 2.3.4.4b has become the dominant circulating lineage worldwide [6]. In March 2024, the B3.13 genotype of H5N1 clade 2.3.4.4b was identified in dairy cattle in the United States and was associated with human infections [5,7]. By the end of 2024, another genotype (D1.1) of H5N1 clade 2.3.4.4b was reported, which also caused infections in both dairy cattle and humans [8]. Notably, the host range of H5N1 clade 2.3.4.4b has continued to expand, with infections reported in a wide range of species, including poultry, wild birds, dairy cattle, foxes, bears, and cats, highlighting its increasing zoonotic potential and its role as a major global public health concern [6,9]. In light of the global persistence and diversification of H5N1 clade 2.3.4.4b, bolstering local surveillance in key regions of China is imperative for effective early warning and risk mitigation.

Jining City, located in northern China, is a major urban center with unique ecological and epidemiological significance. It is home to Weishan Lake, the largest freshwater lake in northern China, and lies along the East Asian–Australasian Flyway, one of the most important migratory bird routes in the world. In addition, Jining is an important hub for poultry breeding and trading in China. However, epidemiological and whole-genome surveillance data regarding the H5N1 clade 2.3.4.4b remain limited in Northern China. To date, no studies have reported on the epidemiology and whole-genome characteristics of H5 subtype avian influenza viruses in Jining and its surrounding areas for the 2024–2025 period. In particular, since the emergence of the B3.13 and D1.1 genotypes of the H5N1 clade 2.3.4.4b lineage in North America, studies focusing on this clade in Northern China have been scarce. To investigate avian influenza virus contamination in poultry-related environments, this study conducted surveillance of environmental samples collected from poultry-associated sites in Jining during 2024–2025. We aimed to characterize the epidemiological patterns and whole-genome features of H5 subtype avian influenza viruses, specifically focusing on epidemiological features, genetic diversity, antigenic site variations, and key amino acid mutations. This study will clarify the molecular evolutionary patterns and potential public health risks of environmental viruses in this region, providing reference data for surveillance, risk assessment, and control strategies.

## 2. Materials and Methods

### 2.1. Sample Collection

Yanzhou, Jinxiang, Zoucheng and Wenshang in Jining City were selected as monitoring sites. Environmental samples associated with poultry were collected in March, June, September, and December of 2024–2025. Sample types included feces, cage surface swabs, chopping boards used for poultry slaughter, poultry washing wastewater, drinking water, and other environmental specimens. A total of 428 samples were collected in 2024 and 320 samples in 2025.

### 2.2. Nucleic Acid Detection

An amount of 200 μL of each sample was used for nucleic acid extraction using an automated nucleic acid extraction system (GeneRotex 96, Tianlong Science and Technology Co., Ltd., Xi’an, China) with the qEx-DNA/RNA Viral Nucleic Acid Extraction Kit (Cat. No. T183).

Detection of influenza A virus and H5/H7/H9 subtype avian influenza viruses was performed using reverse transcription quantitative real-time polymerase chain reaction (RT-qPCR) on a real-time PCR system (Gentier 96, Tianlong, Xi’an, China), with commercial detection kits for influenza A virus (Cat. No. SJ-LG-001-2) and H5/H7/H9 subtypes (Cat. No. SJ-LG-301-2) provided by BioGerm (Qingdao, China).

### 2.3. Whole-Genome Sequencing of H5 Subtype Viruses

Whole-genome capture and amplification of H5 subtype avian influenza viruses were performed using the ULSEN ultra-sensitive influenza A virus whole-genome capture kit (low viral load) (Cat. No. V-090417; Beijing WeFuture Technology Co., Ltd., Beijing, China).

Purification was carried out using VAHTS DNA Clean Beads (Cat. No. N411-01-AA; Vazyme Biotech Co., Ltd., Nanjing, China), and nucleic acid quantification was performed using a Qubit 3 fluorometer (Invitrogen, Boston, MA, USA).

Sequencing libraries were constructed using the VAHTS RNA Multi-PCR Library Prep Kit (Cat. No. NA-211-C4; Vazyme, Nanjing, China), followed by indexing with the Nextera XT Index Kit v2 Set A (Cat. No. 15052163; Illumina, San Diego, CA, USA). Whole-genome sequencing was performed on an Illumina MiSeq platform using the MiSeq Reagent Micro Kit v2 (300 cycles; Cat. No. 15036715; Illumina, San Diego, CA, USA).

### 2.4. Bioinformatics Analysis

Reference sequences of the WHO-recommended H5N1 clade 2.3.4.4b vaccine strain A/Jiangsu/NJ210/2023 (H5N1) (GISAID ID: EPI_ISL_17075747) and the H5N6 clade 2.3.4.4b vaccine strain A/Fujian-Sanyuan/21099/2017 (GISAID ID: EPI_ISL_304404) were downloaded from the GISAID database.

Whole-genome assembly was performed using CLC Genomics Workbench 25 (QIAGEN, Hilden, Germany). Sequence alignment, similarity analysis, and amino acid variation analysis were conducted using MEGA version 7.0.14. Phylogenetic trees were constructed using the maximum-likelihood method with 1000 bootstrap replicates.

Protein tertiary structures were predicted using HelixFold3. N-linked glycosylation sites were predicted using NetNGlyc-1.0, and phosphorylation sites were predicted using NetPhos-3.1.

### 2.5. Statistical Analysis

Statistical analyses were performed using SPSS version 20.0. Differences in positivity rates were compared using the chi-square (*χ*^2^) test. A *p* value < 0.05 was considered statistically significant.

## 3. Results

### 3.1. Epidemiological Characteristics of Avian Influenza Viruses in Jining City, 2024–2025

A total of 748 environmental samples were collected during 2024–2025, among which 120 were positive for influenza A virus, yielding an overall positivity rate of 16.04% (120/748). The positivity rate was significantly higher in 2025 (23.13%, 74/320) than in 2024 (10.75%, 46/428) (*χ*^2^ = 20.83, *p* < 0.001) (Table 1).

The overall positivity rate of H5 subtype avian influenza virus was 8.16% (61/748). Specifically, the positivity rate increased from 5.37% (23/428) in 2024 to 11.88% (38/320) in 2025, with a statistically significant difference between the two years (*χ*^2^ = 10.33, *p* < 0.01) (Table 1).

**Figure 1:**
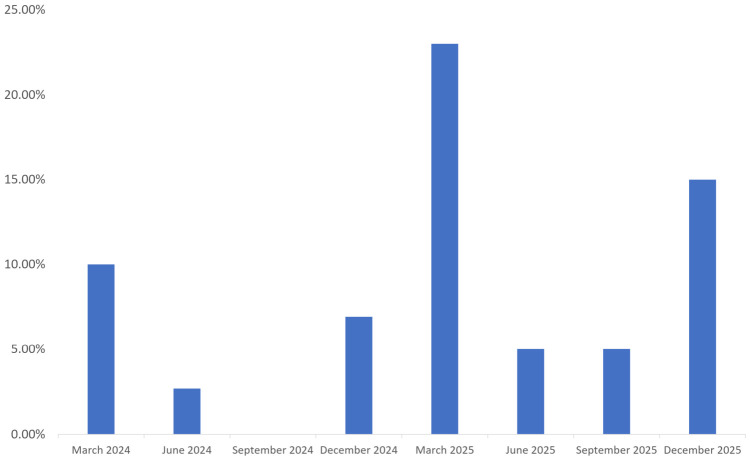
Detection rates of H5 subtype avian influenza viruses in Jining City, 2024–2025. The *x*-axis represents the months, and the y-axis indicates the positivity rate of H5 subtype avian influenza viruses.

Seasonal variation was also observed. The positivity rates of H5 subtype virus were higher in March (17.22%, 31/180) and December (8.54%, 17/199), whereas lower rates were detected in June (3.81%, 11/289) and September (2.50%, 2/80). The differences in positivity rates among months were statistically significant (*χ*^2^ = 30.51, *p* < 0.001) (Table 1; Figure 1). Monitoring results from different sampling sites are shown in Supplementary Table S1.

### 3.2. Whole-Genome Sequencing of H5 Subtype Avian Influenza Viruses in Jining City (2024–2025)

Whole-genome sequencing was performed on H5-positive samples, yielding 12 complete H5 subtype avian influenza virus genomes (two genomes lacked PB2 gene sequences). Among these, five genomes were obtained in 2024, all belonging to the H5N1 subtype, and were designated as *A/Env/shandongjining/1–5/2024 (H5N1)*.

In 2025, seven genomes were obtained, including four H5N1 and three H5N6 subtype viruses, designated as *A/Env/shandongjining/1–4/2025 (H5N1)* and *A/Env/shandongjining/1–3/2025 (H5N6)*, respectively. All sequences were deposited in the GISAID database (accession numbers are provided in Supplementary Table S2).

Clade classification based on HA gene sequences using Nextclade v3.18.1 showed that all 12 strains belonged to clade 2.3.4.4b. Genotype analysis of the nine H5N1 genomes using GenoFLU indicated that all strains were classified as “not assigned,” and no known genotypes such as B3.13 or D1.1 were identified.

### 3.3. Phylogenetic Analysis

**Figure 2:**
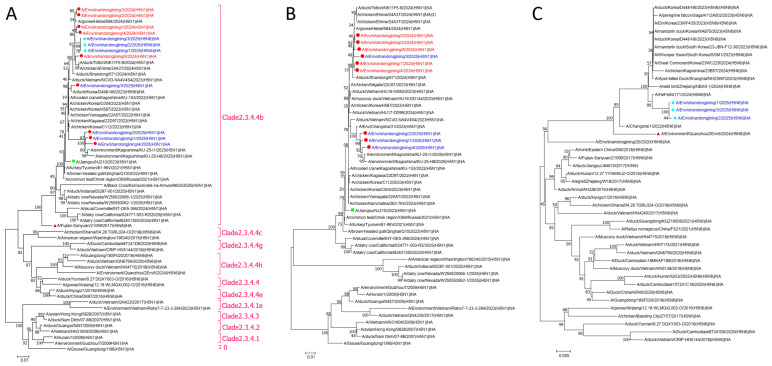
Phylogenetic analysis of HA, N1, and N6 genes of H5 subtype avian influenza viruses in Jining City. (**A**) Phylogenetic tree of the HA gene. (**B**) Phylogenetic tree of the N1 gene. (**C**) Phylogenetic tree of the N6 gene. Phylogenetic trees were constructed using MEGA version 7.0.14 based on the maximum-likelihood method with 1000 bootstrap replicates. █ indicates the WHO-recommended H5N1 clade 2.3.4.4b vaccine reference strain *A/Jiangsu/NJ210/2023 (H5N1)*. ▲ indicates the WHO-recommended H5N6 clade 2.3.4.4b vaccine reference strain *A/Fujian-Sanyuan/21099/2017*. ● indicates H5N1 subtype strains from Jining City, and ● indicates H5N6 subtype strains from Jining City. Red text denotes strains collected in 2024, and blue text denotes strains collected in 2025. Pink brackets and text denote the HA clade.

**Figure 3:**
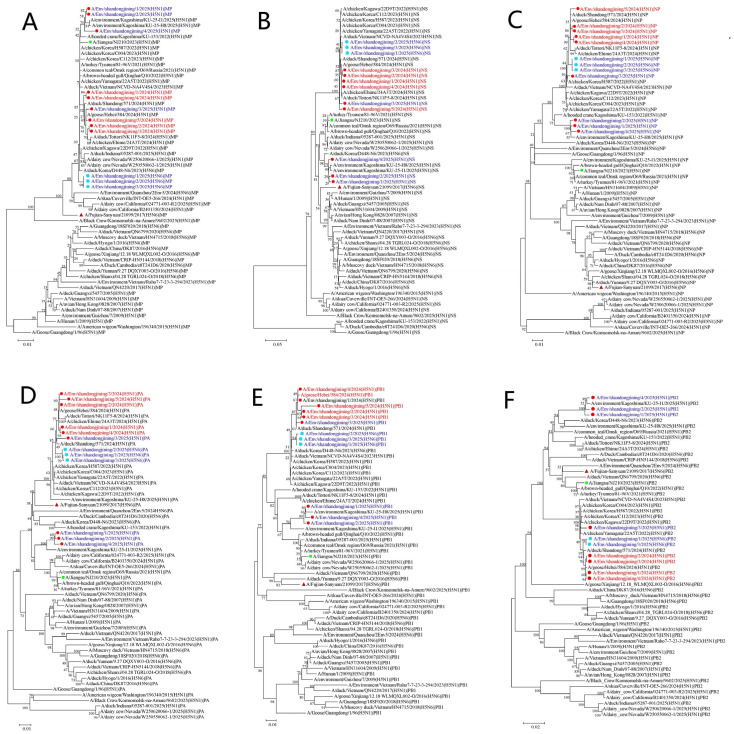
Phylogenetic analysis of MP, NS, NP, PA, PB1, and PB2 genes of H5 subtype avian influenza viruses in Jining City. (**A**) Phylogenetic tree of the MP gene. (**B**) Phylogenetic tree of the NS gene. (**C**) Phylogenetic tree of the NP gene. (**D**) Phylogenetic tree of the PA gene. (**E**) Phylogenetic tree of the PB1 gene. (**F**) Phylogenetic tree of the PB2 gene. Phylogenetic trees were constructed using MEGA version 7.0.14 based on the maximum-likelihood method with 1000 bootstrap replicates. █ indicates the WHO-recommended H5N1 clade 2.3.4.4b vaccine reference strain *A/Jiangsu/NJ210/2023 (H5N1)*. ▲ indicates the WHO-recommended H5N6 clade 2.3.4.4b vaccine reference strain *A/Fujian-Sanyuan/21099/2017*. ● indicates H5N1 subtype strains from Jining City, and ● indicates H5N6 subtype strains from Jining City. Red text denotes strains collected in 2024, and blue text denotes strains collected in 2025.

Phylogenetic trees were constructed for the HA, N1, N6, MP, NS, NP, PA, PB1, and PB2 genes using the 12 H5 subtype genomes from Jining, WHO-recommended vaccine reference strains (*A/Jiangsu/NJ210/2023 [H5N1]* and *A/Fujian-Sanyuan/21099/2017 [H5N6]*), and representative circulating H5N1 and H5N6 strains from recent years (Figure 2 and Figure 3).

In the HA phylogenetic tree, all 12 strains clustered within clade 2.3.4.4b together with the reference vaccine strains. The HA sequences from Jining were more closely related to the H5N1 vaccine strain *A/Jiangsu/NJ210/2023 (H5N1)* and more distantly related to the H5N6 vaccine strain *A/Fujian-Sanyuan/21099/2017* (Figure 2A).

Similarly, in the MP, NS, NP, and PB1 phylogenetic trees, all 12 strains showed closer genetic relationships to the H5N1 vaccine reference strain (Figure 3A–E). In contrast, in the PA phylogenetic tree, all strains clustered more closely with the H5N6 vaccine reference strain *A/Fujian-Sanyuan/21099/2017* (Figure 3D).

### 3.4. Sequence Similarity Analysis

Compared with the WHO-recommended H5N1 clade 2.3.4.4b vaccine strain *A/Jiangsu/NJ210/2023 (H5N1)*, the nucleotide sequence identities of the H5 subtype avian influenza viruses from Jining (2024–2025) were as follows: HA (97.24–98.71%), N1 (97.26–98.56%), MP (98.64–99.12%), NS (90.73–95.79%), NP (92.91–93.93%), PA (94.64–95.54%), PB1 (91.88–94.25%), and PB2 (92.23–92.36%).

**Figure 4:**
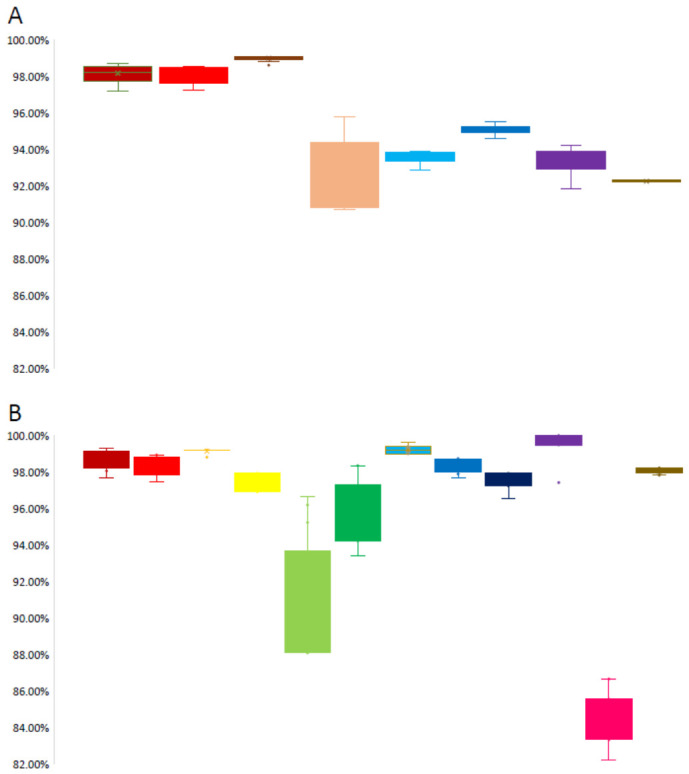
Sequence similarity analysis of genomic nucleotide sequences and encoded protein amino acid sequences of H5 subtype avian influenza viruses in Jining City, 2024–2025. (**A**) Nucleotide sequence similarity of HA, N1, MP, NS, NP, PA, PB1, and PB2 genes compared with the WHO-recommended H5N1 clade 2.3.4.4b vaccine strain *A/Jiangsu/NJ210/2023 (H5N1)*. (**B**) Amino acid sequence similarity of HA, N1, M1, M2, NS1, NEP, NP, PA, PA-X, PB1, PB1-F2, and PB2 proteins compared with *A/Jiangsu/NJ210/2023 (H5N1)*. █ HA, █ N1, █ MP, █ M1, █ M2, █ NS, █ NS1, █ NEP, █ NP, █ PA, █ PA-X, █ PB1, █ PB1-F2, █ PB2. Solid circles denote outlying observations.

At the amino acid level, sequence identities were observed as follows: HA (97.70–99.29%), N1 (97.44–98.93%), M1 (98.81–99.21%), M2 (96.91–97.94%), NS1 (88.10–96.67%), NEP (93.39–98.35%), NP (99.00–99.60%), PA (97.68–98.73%), PA-X (96.55–97.93%), PB1 (97.42–100%), PB1-F2 (82.22–86.67%), and PB2 (97.82–98.23%) (Figure 4).

### 3.5. Amino Acid Mutation Analysis

The hemagglutinin (HA) protein is the major antigenic determinant and receptor-binding protein of influenza viruses. In H5 subtype viruses, antigenic epitopes are distributed across five regions (sites 1–5), including sites 1 (114–142 aa), 2 (151–156, 182–190, and 192–195 aa), 3 (39–40, 43–44, 267–273, and 275 aa), 4 (163, 197–204, 210, 212–216, 218–223, and 238 aa), and 5 (53–54, 66, 68–74, and 82–85 aa) [10,11].

Compared with the vaccine strain *A/Jiangsu/NJ210/2023 (H5N1)*, a total of 16 amino acid substitutions were identified in the HA sequences of the 12 strains. Mutations located in antigenic site 1 included L115Q (25.00%), S120N (25.00%), and N124D (25.00%), while mutations in antigenic site 2 included D154N (8.33%), N183D (25.00%), T188K (25.00%), and N189K (100%). Notably, two strains harbored six substitutions and one strain harbored seven substitutions within antigenic epitopes, suggesting antigenic drift.

The receptor-binding regions of H5 HA include the 130-loop (131–134 aa), 150-loop (151–159 aa), 190-helix (189–194 aa), and 220-loop (217–224 aa). Mutations Q222L and G224S have been reported to shift receptor binding preference from avian to human-type receptors [11,12]. In this study, mutations D154N (8.33%), A156E (8.33%), and N189K (100%) were identified within receptor-binding regions. Residue 189 is known to influence affinity for avian-type (α-2,3-linked) sialic acid receptors [13]. However, no Q222L or G224S mutations were detected.

The neuraminidase (NA) protein contains conserved catalytic residues (R118, D151, R152, R224, E276, R292, R371, and Y406) and framework residues (E119, R156, W178, S179, D198, I222, E227, E277, D293, and E425). Although 19 amino acid substitutions were identified in the N1 sequences, none occurred in these key functional sites.

In the matrix proteins, mutations D30N and A215T in M1 have been associated with reduced virulence [14]. In this study, three substitutions (I42L, S116A, and T121P) were observed, while no mutations at positions 30 or 215 were detected. The M2 protein residue L26 has been associated with viral replication efficiency [15], and mutations L26I, V27A, and S31N confer resistance to amantadine and rimantadine [16]. None of these resistance-associated mutations were identified in the Jining strains.

**Figure 5:**
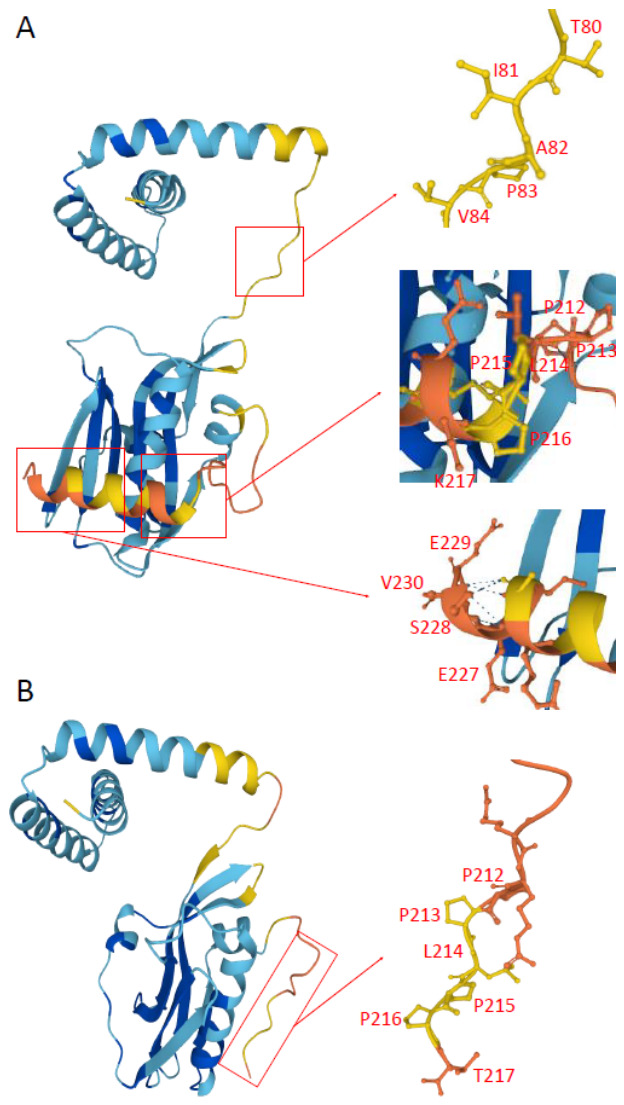
Structural analysis of the NS1 protein of H5 subtype avian influenza viruses. (**A**) Predicted structure of NS1 encoded by the H5N1 clade 2.3.4.4b vaccine strain A/Jiangsu/NJ210/2023 (H5N1), containing the TIAPV (80–84) motif, the SH3-binding motif PPLPPK (212–217), and the C-terminal PDZ-binding motif ESEV (227–231). (**B**) Predicted structure of NS1 from the Jining strain A/Env/shandongjining/2/2024 (H5N1). The TIAPV (80–84) motif is absent, the SH3-binding motif is altered from PPLPPK to PPLPPT (212–217), and the C-terminal PDZ-binding motif ESEV (227–231) is truncated. Colors represent model confidence based on predicted Local Distance Difference Test (pLDDT) scores: █ very high confidence (pLDDT > 90), █ high confidence (pLDDT > 70), █ low confidence (50 < pLDDT ≤ 70), and █ very low confidence (pLDDT ≤ 50).

NS1 is a critical virulence factor. Mutations such as D92E, P42S, S228P, G205R, A149V, L144A, and L146A and deletions at positions 80–84 have been reported to affect virulence [17,18,19,20]. In this study, all NS1 sequences exhibited an 80–84 deletion (Figure 5A). Residues 212–217 form the SH3-binding domain, and K217 is essential for interaction with SH3-domain-containing proteins [21]. Mutations P213S and K217T identified in this study may impair such interactions (Figure 5A). Additionally, the C-terminal ESEV motif mediates binding to PDZ-domain-containing proteins [22]. The Q218 stop mutation observed in 75.00% of strains resulted in loss of this motif (Figure 5B). No pathogenicity-associated mutations were detected in NEP [19].

In NP, mutations such as N319K, Q357K, and K470R have been associated with increased virulence and mammalian adaptation, whereas K91R and K198R reduce replication [23,24,25,26,27]. None of these mutations were observed.

The viral polymerase complex (PA, PB1, and PB2) plays a central role in viral replication [28]. Several PA mutations (e.g., K142E, P224S, N383D, and I550L) enhance replication, whereas others reduce replication or affect virulence [29,30,31,32]. In this study, all PA sequences contained replication-associated residues S224 and L550, and some contained D383.

PB1 mutations such as L13P enhance replication, whereas others reduce virulence [33,34,35]. All strains contained P13 and L598.

PB2 mutations including D256G, I504V, T588I, E627K, and D701N enhance replication, while K482R increases virulence and R368K, D701N, and S714I/R enhance mammalian adaptation [26,36,37,38,39,40,41]. All PB2 sequences contained V504, but no E627K or D701N mutations were detected. Host-adaptive mutations reported in other species (e.g., T271A in minks, V478I in ferrets, K526R in cats, Q591K in marine mammals, and M631L in cattle) were also absent [42].

**Figure 6:**
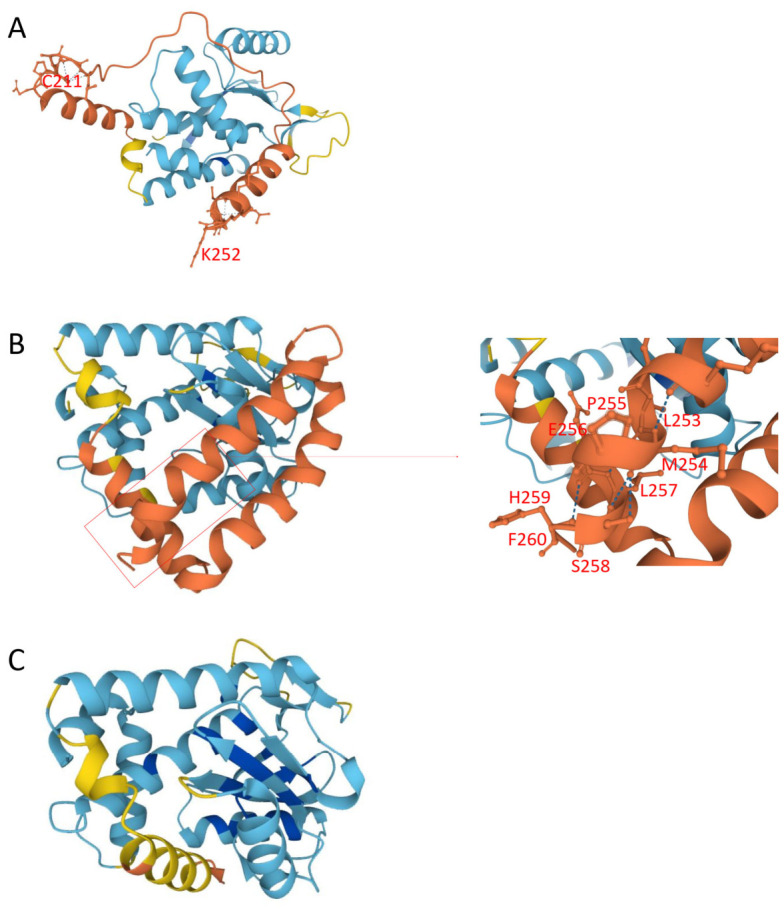
Structural analysis of the PA-X protein of H5 subtype avian influenza viruses. The tertiary structure of PA-X was predicted using HelixFold3. (**A**) Predicted structure of PA-X encoded by the H5N1 clade 2.3.4.4b vaccine strain *A/Jiangsu/NJ210/2023 (H5N1)*. (**B**) Predicted structure of PA-X from the Jining strain *A/Env/shandongjining/2/2024 (H5N1)*, showing a C-terminal extension with an additional LMPELSHF (253–260) amino acid sequence. (**C**) Predicted structure of PA-X from the Jining strain *A/Env/shandongjining/3/2025 (H5N1)*, harboring a C211 stop mutation that results in truncation and loss of the C-terminal region (amino acids 211–252). Colors indicate model confidence based on predicted Local Distance Difference Test (pLDDT) scores: █ very high confidence (pLDDT > 90), █ high confidence (pLDDT > 70), █ low confidence (50 < pLDDT ≤ 70), and █ very low confidence (pLDDT ≤ 50).

PA-X contains key residues for host shutoff activity, including positions 192–206 and the C-terminal region (233–252 aa) [43,44,45,46]. Mutations K198Q, R199K, K203R, and K206R were identified within these functional regions. A C211 stop mutation led to truncation of the C-terminal domain, while in three strains, stop codon alterations resulted in a C-terminal extension (LMPELSHF) (Figure 6). Additional mutations such as Y24H and N204D may also affect PA-X function.

PB1-F2 residues 68–71 are critical for protein stability, and mutations such as D70V and S71F enhance interferon antagonism [47,48]. In this study, mutations D70G, D70E, and S71F were identified within this region.

### 3.6. Glycosylation and Phosphorylation Site Analysis

N-linked glycosylation sites in H5N1 viral proteins were predicted using NetNGlyc-1.0. Compared with the reference strain, the NS1 sequences of the Jining isolates exhibited the acquisition of a glycosylation site at positions NIT127–129. Additionally, NEP and PB2 gained glycosylation sites at NGT62–64 and NGS334–336, respectively. In contrast, PA-X lost the NRT237–239 glycosylation site (Table 2).

Phosphorylation sites were predicted using NetPhos-3.1. In the HA protein, the loss of the S120 phosphorylation site was identified within an antigenic region. In NS1, a novel phosphorylation site at S213 was detected within the SH3-binding domain, which may influence host protein interactions (Table 2).

Phosphorylation of NP at residue S482 has been reported to promote NP oligomerization, enhance viral ribonucleoprotein (vRNP) assembly, and facilitate viral replication [49]. In this study, an S482N mutation (8.33%) resulted in the loss of this phosphorylation site (Table 2).

Phosphorylation is also critical for PB1 function. Loss of phosphorylation at residue S384 has been shown to moderately reduce viral gene expression [23]. A mutation (S384P, 25.00%) observed in PB1 resulted in the loss of this phosphorylation site (Table 2).

## 4. Discussion

Marked geographic differences in the positivity rates of H5 subtype avian influenza viruses have been reported between southern and northern China. During 2019–2023, the positivity rate in northern China was approximately 1.01%, whereas it reached 7.90% in southern China [50]. Jining City, located in northern China, showed a positivity rate of 1.54% during 2018–2023 [51], which was higher than the overall northern China level during the same period. In the present study, a total of 61 H5 subtype influenza virus-positive samples were detected among 748 environmental specimens during 2024–2025, exceeding historical levels in Jining. Notably, a total of 46 H5 subtype avian influenza virus-positive samples were detected among 428 environmental samples tested in 2024, and 74 positive samples among 320 samples tested in 2025, indicating a significant upward trend in H5 virus circulation intensity.

A total of 12 H5 subtype avian influenza virus genomes were obtained, all of which belonged to clade 2.3.4.4b, consistent with the currently dominant global lineage. The co-circulation of both H5N1 and H5N6 subtypes in Jining indicates increased viral diversity and highlights the complexity of local epidemiology. Although no B3.13 or D1.1 genotypes were identified, these genotypes have been widely reported in North America and have infected humans and mammals such as dairy cattle [52]. However, the northern region of North America also lies along the East Asian-Australasian Flyway, there is a potential risk of the B3.13 and D1.1 genotypes spreading via migratory birds. Therefore, continued surveillance of H5 viruses remains essential.

The HA protein is the primary determinant of antigenicity and receptor binding, and its amino acid substitutions directly influence antigenic properties and host range. Antigenic drift is generally defined by the presence of ≥4 amino acid substitutions across at least two antigenic sites [53]. In this study, three H5N1 strains from 2025 exhibited 6–7 substitutions across multiple antigenic sites, suggesting reduced antigenic matching with the vaccine strain *A/Jiangsu/NJ210/2023 (H5N1)*. Importantly, no human-adaptive mutations such as Q222L or G224S were detected, indicating that the current strains likely retain avian receptor specificity and pose a relatively low immediate risk for human infection.

Non-structural proteins, including NS1, PA-X, and PB1-F2, are critical virulence factors that modulate host immune responses through interferon antagonism and host shutoff activity. NS1 mutations such as P213S and Q218 stop may disrupt interactions with SH3- and PDZ-domain-containing host proteins, potentially reducing virulence. Similarly, mutations in PA-X (e.g., K198Q, R199K, K203R, and K206R) located within key functional domains may impair host shutoff activity [44]. The C211 stop mutation further truncates the C-terminal region, which is essential for PA-X function. In contrast, mutations in PB1-F2 (D70G, D70E, and S71F) may enhance protein stability and strengthen inhibition of interferon signaling, potentially contributing to immune evasion.

The viral polymerase complex (PA, PB1, and PB2) plays a central role in viral replication and transcription. The presence of replication-associated residues (S224, L550, and D383 in PA; P13 in PB1; and V504 in PB2) suggests maintained or enhanced replication capacity of circulating strains. However, the absence of key mammalian adaptation markers such as E627K and D701N in PB2 indicates that these viruses have not yet acquired strong adaptation to mammalian hosts.

In summary, this study systematically elucidates the prevalence characteristics and whole-genome variation patterns of H5 subtype avian influenza viruses in Jining City during 2024–2025. The prevalence of H5 subtype avian influenza viruses in Jining increased markedly during 2024–2025. The circulating strains were predominantly clade 2.3.4.4b, with co-circulation of H5N1 and H5N6 subtypes, evidence of antigenic drift, and characteristic molecular variations, including the K217T mutation and Q218 stop mutation in the NS1 protein, as well as the C218 mutation and C-terminal extension (LMPELSHF) in the PA-X protein. However, given that Jining is situated along the East Asian-Australasian Flyway, there remains a risk of introducing overseas H5N1 avian influenza genotypes. These findings highlight the need to strengthen surveillance, particularly during high-risk seasons and in key ecological regions, and to enhance early warning and immunization strategies. Continuous monitoring is essential to detect potential reassortment events and emerging mutations that may increase public health risk. This study provides important data for avian influenza prevention and control in Jining and offers valuable insights for other regions located along major migratory bird flyways.

## Figures and Tables

**Table 1:** Detection results of avian influenza viruses in Jining City, 2024–2025.

**Year**	**Month**	**Sample Size**	**Influenza A**		**H5**		**H9**		**H5 + H9**		**Unsubtyped**	
			**P (*n*)**	**PR (%)**	**P (*n*)**	**PR (%)**	**P (*n*)**	**PR (%)**	**P (*n*)**	**PR (%)**	**P (*n*)**	**PR (%)**
2024	March	80	14	17.50%	8	10.00%	6	7.50%	0	0.00%	0	0.00%
	June	149	13	8.72%	4	2.68%	9	6.04%	0	0.00%	0	0.00%
	September	40	2	5.00%	0	0.00%	2	5.00%	0	0.00%	0	0.00%
	December	159	17	10.69%	4	2.52%	6	3.80%	7	4.43%	0	0.00%
2025	March	100	42	42.00%	18	18.00%	14	14.00%	5	5.00%	5	5.00%
	June	140	14	10.00%	6	4.29%	6	4.29%	1	0.71%	1	0.71%
	September	40	8	20.00%	2	5.00%	2	5.00%	0	0.00%	4	10.00%
	December	40	10	25.00%	6	15.00%	4	10.00%	0	0.00%	0	0.00%
**Total**		748	120	16.04%	48	6.42%	49	6.55%	13	1.74%	10	1.34%

P: positive; PR: positive rate.

**Table 2:** Analysis of glycosylation and phosphorylation sites in H5 subtype avian influenza virus proteins from Jining City.

**Protein**	**Glycosylation Sites**		**Phosphorylation Sites**	
	**Gain**	**Loss**	**Gain**	**Loss**
M1	-	-	-	S116 (100%)
M2	-	-	S25 (8.33%)	-
NS1	NIT127-129 (91.67%)	-	S48 (100%), T129 (100%), T202 (75.00%), S213 (8.33%)	T80 (100%), S114 (75.00%)
NEP	NGT62-64 (75.00%)	-	T64 (75.00%)	S60 (75.00%)
NP	-	-	S343 (25.00%), S473 (8.33%)	T373 (66.67%), S482 (8.33%)
PA	-	-	S212 (8.33%), S400 (25.00%)	Y24 (8.33%), T173 (8.33%), T210 (8.33%)
PA-X	-	NRT237-239 (8.33%)	-	Y24 (8.33%), T173 (8.33%), S219 (75.00%)
PB1	-	-	T577 (8.33%)	S261 (8.33%), S384 (25.00%), S654 (33.33%)
PB1-F2	-	-	-	T10 (8.33%), S63 (8.33%), S71 (41.67%), S82 (75.00%)
PB2	NGS334-336 (70.00%)	-	S590 (70.00%), S639 (30.00%), T676 (100%), S698 (30.00%)	T129 (30.00%), S334 (70.00%), S715 (100%)

## Data Availability

The original data presented in the study are openly available in GISAID. The accession numbers (GISAID IDs) are listed in the Supplementary Material of this article.

## Supplementary Materials

The following supporting information can be downloaded at https://www.mdpi.com/article/10.3390/pathogens15050521/s1, Table S1: Monitoring results from different sampling sites; Table S2: GISAID accession numbers of 12 H5 subtype avian influenza virus sequences in Jining City.
