# Supplementary material for: Epidemiological and Genomic Characterization of H5 Subtype Avian Influenza Viruses in Jining City, 2024–2025
Source: Pathogens. 2026 May 12;15(5):521. doi: 10.3390/pathogens15050521 (PMC13209583; doi:10.3390/pathogens15050521)
Supplement: Supplementary file 1 [file pathogens-15-00521-s001.zip › Supplementary Table S1 Monitoring results from different sampling sites.pdf]

Supplementary Table S1. Monitoring results from different sampling sites

Year	County	Sample size	Influenza A		H5		H9		H5+H9		Unsubtyped	
			P (*n*)	PR（%）	P (*n*)	PR（%）	P (*n*)	PR（%）	P (*n*)	PR（%）	P (*n*)	PR（%）
2024	Yanzhou	114	4	3.51%	0	0.00%	4	3.51%	0	0.00%	0	0.00%
	Jinxiang	105	9	8.57%	4	3.81%	5	4.76%	0	0.00%	0	0.00%
	Zoucheng	105	27	25.71%	10	9.52%	10	9.52%	7	6.67%	0	0.00%
	Wenshang	104	6	5.77%	2	1.92%	4	3.85%	0	0.00%	0	0.00%
2025	Yanzhou	75	9	12.00%	2	2.67%	5	6.67%	1	1.33%	1	1.33%
	Jinxiang	80	12	15.00%	1	1.25%	4	5.00%	0	0.00%	7	8.75%
	Zoucheng	85	30	35.29%	13	15.29%	12	14.12%	3	3.53%	2	2.35%
	Wenshang	80	23	28.75%	16	20.00%	5	6.25%	2	2.50%	0	0.00%
**Total**		748	120	16.04%	48	6.42%	49	6.55%	13	1.74%	10	1.34%
