# Supplementary material for: Epidemiological and Genomic Characterization of H5 Subtype Avian Influenza Viruses in Jining City, 2024–2025
Source: Pathogens. 2026 May 12;15(5):521. doi: 10.3390/pathogens15050521 (PMC13209583; doi:10.3390/pathogens15050521)
Supplement: Supplementary file 1 [file pathogens-15-00521-s001.zip › Supplementary Table S2 GISAID accession numbers of 12 H5 subtype avian influenza virus sequences in Jining City.pdf]

Supplementary Table S2. GISAID accession numbers of 12 H5 subtype avian influenza virus sequences in Jining City.

Isolate Name	Collection Date	GISAID Id
A/Env/shandongjining/1/2024(H5N1)	2024-06-28	EPI_ISL_20362428
A/Env/shandongjining/3/2024(H5N1)	2024-06-28	EPI_ISL_20363574
A/Env/shandongjining/2/2024(H5N1)	2024-06-28	EPI_ISL_20363575
A/Env/shandongjining/4/2024(H5N1)	2024-06-28	EPI_ISL_20363576
A/Env/shandongjining/1/2025(H5N1)	2025-03-27	EPI_ISL_20363577
A/Env/shandongjining/5/2024(H5N1)	2024-12-25	EPI_ISL_20363579
A/Env/shandongjining/2/2025(H5N1)	2025-03-28	EPI_ISL_20363580
A/Env/shandongjining/3/2025(H5N1)	2025-06-23	EPI_ISL_20363582
A/Env/shandongjining/4/2025(H5N1)	2025-12-22	EPI_ISL_20363587
A/Env/shandongjining/1/2025(H5N6)	2025-03-27	EPI_ISL_20363589
A/Env/shandongjining/2/2025(H5N6)	2025-03-27	EPI_ISL_20363632
A/Env/shandongjining/3/2025(H5N6)	2025-03-27	EPI_ISL_20363633
